# DEPTOR regulates osteogenic differentiation via inhibiting MEG3-mediated activation of BMP4 signaling and is involved in osteoporosis

**DOI:** 10.1186/s13287-018-0935-9

**Published:** 2018-07-04

**Authors:** Si Chen, Lingfei Jia, Shan Zhang, Yunfei Zheng, Yongsheng Zhou

**Affiliations:** 1 Department of Prosthodontics, Peking University School and Hospital of Stomatology, 22 Zhongguancun South Avenue, Haidian District, Beijing, 100081 China; 2 Department of Oral and Maxillofacial Surgery, Peking University School and Hospital of Stomatology, 22 Zhongguancun South Avenue, Haidian District, Beijing, 100081 China; 3 Central Laboratory, Peking University School and Hospital of Stomatology, 22 Zhongguancun South Avenue, Haidian District, Beijing, 100081 China; 4 Department of Orthodontics, Peking University School and Hospital of Stomatology, 22 Zhongguancun South Avenue, Haidian District, Beijing, 100081 China; 5 National Engineering Lab for Digital and Material Technology of Stomatology, Peking University School and Hospital of Stomatology, 22 Zhongguancun South Avenue, Haidian District, Beijing, 100081 China; 6 National Clinical Research Center for Oral Diseases, 22 Zhongguancun South Avenue, Haidian District, Beijing, 100081 China

**Keywords:** DEP domain containing mTOR interacting protein, Osteoporosis, Osteogenesis, Maternally expressed 3 (nonprotein coding), Bone morphogenetic protein 4, Bone engineering

## Abstract

**Background:**

The mammalian target of rapamycin (mTOR) pathway plays a significant role in osteogenic differentiation and bone maintenance. As the only known endogenous inhibitor of mTOR function, DEP domain containing mTOR interacting protein (DEPTOR) is potentially involved in stem cell differentiation, although the pathophysiological significance and its molecular mechanisms remain unclear. The present study aimed to elucidate the effects of DEPTOR on the progress of osteoporosis and investigate the underlying molecular mechanisms of osteogenic regulation.

**Methods:**

An ovariectomy mouse model with decreased bone formation and osteogenic induction with bone marrow mesenchymal stem cells (BMSCs) were used to investigate the relationship between DEPTOR and osteogenic events. A loss-of-function investigation was then performed to explore the role of DEPTOR in the osteogenic differentiation of BMSCs both in vitro and in vivo. Finally, long noncoding RNA (lncRNA) and mRNA sequences were investigated to reveal the underlying mechanisms of DEPTOR in osteogenic regulation. RNA interference, western blotting, and chromatin immunoprecipitation assays were performed for further mechanistic determination.

**Results:**

The results indicated that DEPTOR contributes to the progress of osteoporosis, and higher expression of Deptor was observed in osteoporotic bones. The expression of DEPTOR was reduced during the osteogenic differentiation of BMSCs, and knockdown of *DEPTOR* promoted BMSC osteogenesis in vitro and in vivo. lncRNA and mRNA sequences indicated that knockdown of *DEPTOR* upregulated the expression of maternally expressed 3 (nonprotein coding) (*MEG3*), which subsequently activated bone morphogenetic protein 4 (BMP4) signaling. Furthermore, DEPTOR could bind to a specific region (− 1000 bp ~ 0) of the *MEG3* promoter to regulate its transcription, and inhibition of *MEG3* reduced BMP4 activation triggered by *DEPTOR* knockdown.

**Conclusions:**

Taken together, our study revealed a novel function of DEPTOR in osteogenic differentiation by inhibiting MEG3-mediated activation of BMP4 signaling, which suggested that DEPTOR could be a therapeutic target for bone loss diseases and skeletal tissue regeneration.

**Electronic supplementary material:**

The online version of this article (10.1186/s13287-018-0935-9) contains supplementary material, which is available to authorized users.

## Background

Osteoporosis is one of the most severe skeletal diseases in the elderly population and postmenopausal women [1]. It is characterized by decreased bone mineral density and destruction of the microarchitectural bone structure, resulting in higher susceptibility to bone fracture [2, 3]. Bone marrow mesenchymal stem cells (BMSCs) are multipotent progenitor cells that can be differentiated into osteoblastic, adipogenic, and chondrogenic lineages [4]. Accumulating research indicates that the aberrant lineage differentiation of endogenous BMSCs contributes to osteoporosis [5–7]. However, the molecular mechanisms by which BMSCs undergo committed osteogenic differentiation remain unclear, hindering the clinical treatment of osteoporosis.

As a serine/threonine kinase that responds to extracellular signaling, the mammalian target of rapamycin (mTOR) is a critical modulator of cell differentiation and tissue regeneration [8, 9]. Emerging studies have demonstrated that mTOR signaling plays a critical role in the osteogenic differentiation of MSCs [10–12]. mTORC1 is recognized as a common effector that mediates bone-related signaling, such as via WNT and bone morphogenetic protein (BMP) [9]. In addition, recent genetic research in mice indicated that impairment of mTORC1 function resulted in increased osteogenesis, while impairment of mTORC2 function led to reduced osteogenesis [12].

DEP domain containing mTOR interacting protein (DEPTOR) is a pivotal protein belonging to the mTORC1 and mTORC2 complexes [13, 14]. As an endogenous mTOR inhibitor, DEPTOR participates in multiple biological events, such as cell growth, apoptosis, autography, and cell differentiation [15]. DEPTOR could act as a stemness factor that plays an important role in the pluripotent maintenance of embryonic stem cells (ESCs). Knockdown of *DEPTOR* promoted ESC differentiation with a corresponding increase of mTORC1 activity [16]. Moreover, a recent study indicated that DEPTOR promoted adipogenic differentiation via activation of the AKT/PKB-PPAR-γ pathway [17]. Our previous research revealed that DEPTOR could function as a negative regulator in miR-375-mediated osteogenesis through feedback activation of IRS1/PI3K/AKT signaling [18].

Given the significance of DEPTOR in osteogenic/adipogenic regulation, the role and function of DEPTOR in bone metabolism and tissue engineering requires further study. In the present study, we demonstrated that DEPTOR participated in the progress of osteoporosis, and higher expression of Deptor was observed in osteoporotic bones. The expression of DEPTOR was reduced during the osteogenic differentiation of BMSCs, and knockdown of *DEPTOR* promoted BMSC osteogenesis, not only in vitro but also in vivo. Furthermore, long noncoding RNA (lncRNA) and mRNA sequencing indicated that reduction of DEPTOR upregulated the expression of maternally expressed 3 (nonprotein coding) (*MEG3*), which subsequently activated bone morphogenetic protein 4 (BMP4)/SMAD1/5/8 signaling, suggesting that DEPTOR could be a therapeutic target for bone loss diseases and skeletal tissue regeneration.

## Methods

### Cell culture and osteogenic induction

All materials were obtained from Sigma-Aldrich (St. Louis, MO, USA) unless otherwise stated. Minimum essential medium alpha (α-MEM), fetal bovine serum (FBS), and 100× antibiotics were purchased from Gibco (Grand Island, NY, USA). Primary human bone marrow mesenchymal stem cells (hBMSCs) from three different healthy donors were obtained from ScienCell (7500; San Diego, CA, USA). Cells between passages 3 and 5 were utilized for the experiments and all of the in-vitro experiments were repeated in triplicate. hBMSCs were cultured in proliferation medium (PM) containing fresh α-MEM, 10% (v/v) FBS, and 1% (v/v) antibiotics at 37 °C in an incubator with an atmosphere consisting of 95% air and 5% CO_2_, with 100% relative humidity. For osteogenic induction, hBMSCs were cultured in osteogenic medium (OM), consisting of fresh α-MEM, 10% (v/v) FBS, 1% (v/v) antibiotics, 10 mmol·L^− 1^ β-glycerophosphate, 0.2 mmol·L^− 1^ l-ascorbic acid, and 100 nmol·L^− 1^ dexamethasone.

### Lentivirus infection

Recombinant lentiviruses expressing short hairpin RNAs targeting *DEPTOR* (shDEPTOR #1, shDEPTOR #2) and the scrambled control (shNC) were purchased from GenePharma (Shanghai, China); the sequences are presented in Additional file 1: Table S1. hBMSCs were exposed with the viral supernatant at a multiplicity of infection of 100 together with polybrene (5 μg·ml^− 1^) for 24 h. Puromycin at 1 μg·ml^− 1^ was then added to the culture medium for at least 3 days to establish stable cell lines.

### RNA sequencing

Total RNAs of each sample were extracted using TruSeq RNA sample preparation reagents (Illumina, San Diego, CA, USA) according to the manufacturer’s instructions, with fragmentation for 4 min at 94 °C. The amplified fragmented cDNA ~ 300 bp in size were sequenced in paired-end mode using an Illumina HiSeq instrument, and two FASTQ files were generated for each sample. The alignment of the reads onto the reference genome and splice site identification were performed using Bowtie/TopHat, with mapping allowing up to two mismatches. The aligned reads were then assembled into transcripts using Cufflinks software, and then computed as normalized values termed fragments per kilobase of exon per million fragments mapped (FPKM). Statistical analysis of differentially expressed genes and transcript splice variants was performed using Cuffdiff, with a false discovery rate (FDR) of 5%.

### Ovariectomy operation of mice

Eight-week-old female C57BL/6 mice were purchased from the Jackson Laboratory (Bar Harbor, ME, USA) and maintained in pathogen-free facilities on a 12-h light/dark cycle. Bilateral ovariectomy (OVX) or sham operation (*N* = 10 per group) was conducted as described previously [19]. The mice were sacrificed by CO_2_ asphyxiation 3 months after surgery. Femurs from the sham or OVX mice were dissected free of soft tissue, and analyzed using high-resolution Inveon microtomography (Siemens, Munich, Germany) after fixation in 4% paraformaldehyde. Images were acquired at an effective pixel size of 8.82 μm, a voltage of 80 kV, a current of 500 μA, and an exposure time of 1500 ms in each of the 360 rotational steps. Parameters of the trabecular region (1–2 mm distal to the proximal epiphysis), including bone volume/total volume (BV/TV), trabecular bone thickness (Tb.Th), and trabecular bone number (Tb.N), were measured using an Inveon Research Workplace (Siemens) according to the guidelines set by the American Society for Bone and Mineral Research (ASBMR). For histological analysis, the femurs were decalcified with 10% EDTA (pH 7.4), dehydrated, and then embedded in paraffin. Slices (5-μm thick) were prepared and stained with hematoxylin and eosin (H&E).

### Immunofluorescence staining of mouse femurs

After fixation in 4% paraformaldehyde at 4 °C overnight, femurs of the sham or OVX mice were decalcified in 14% EDTA (pH 7.4) at room temperature, and then washed in phosphate-buffered saline (PBS) for 2 h. After soaking in 30% sucrose with constant agitation at 4 °C overnight, the femurs were embedded in 22-oxa-1,25-dihydroxyvitamin D_3_ optimum cutting temperature (OCT) compound, and cut into 30-μm thick sections. The primary antibody against Deptor (1:200 dilution; Proteintech, Chicago, IL, USA) was incubated with the slices overnight at 4 °C. Alexa Fluor-546-labeled-goat anti-rabbit IgG (1:1000 dilution; Invitrogen, Carlsbad, CA, USA) was utilized as the secondary antibody, and cell nuclei were counterstained with 2-(4-amidinophenyl)-1H-indole-6-carboxamidine (DAPI) (Invitrogen).

### Isolation of primary mBMSCs

The ends of the femurs from sham and OVX mice were removed with scissors, and the bone marrow was harvested by inserting a syringe needle (27 gauge) into one end of the femur and flushing with α-MEM. After centrifuging at 1000 rpm for 5 min, the cells were collected and cultured in fresh α-MEM mixed with 20% (v/v) FBS and 1% (v/v) antibiotics. The medium was replaced every 2 days to remove the nonadherent cells until full confluency was obtained, and the mouse bone marrow mesenchymal stem cells (mBMSCs) at the first passage were used for the in-vitro experiments to analyze the expression of Deptor in osteoporotic conditions.

### RNA interference

Short interfering (si)RNAs targeting MEG3 (si-MEG3 #1, si-MEG3 #2) and the scramble control (si-NC) were obtained from GenePharma, and the sequences are presented in Additional file 1: Table S1. Transfection of hBMSCs was conducted with Lipofectamine 3000 (Invitrogen) according to the manufacturer’s instructions. Cells were harvested 48 h after transfection for RNA and protein analyses. As for the osteogenic induction, transfection was reconducted every 5 days to guarantee the knockdown efficiency, and cells were collected 7 or 14 days after osteogenic differentiation.

### Chemical administration

Recombinant human Noggin (6057-NG) was purchased from R&D Systems Inc. (Minneapolis, MN, USA). Noggin was reconstituted in sterile PBS containing 0.1% bovine serum albumin (BSA) and administrated at 500 ng·ml^− 1^ according to previous studies [20, 21].

### Alkaline phosphatase staining and quantification

Cells were seeded in six-well plates, and cultured in PM or OM for 7 days. Alkaline phosphatase (ALP) staining was conducted using a nitro-blue tetrazolium chloride (NBT)/5-bromo-4-chloro-3-indolylphosphate toluidine (BCIP) staining kit (CoWin Biotech, Beijing, China) after fixation in 95% ethanol at room temperature. ALP activity was measured using an ALP assay kit (Nanjing Jiancheng Bioengineering Institute, Nanjing, China) according to the manufacturer’s instructions, and normalized to the total protein contents as determined using the BCA method (Thermo Fisher Scientific, Rockford, IL, USA).

### Alizarin red S staining and quantification

Cells were seeded in six-well plates, and cultured in PM or OM for 14 days. Alizarin red S (ARS) staining was performed with 1% ARS (pH 4.2) after fixation in 4% paraformaldehyde. To quantify the degree of mineralization, the stains were dissolved in 100 mmol·L^− 1^ cetylpyridinium chloride and the absorbance was measured at 562 nm.

### von Kossa staining

Cells were seeded in six-well plates, and cultured in PM or OM for 21 days. The cells were fixed incubated with 5% silver nitrate for 30 min in a dark room after fixation in 4% paraformaldehyde, and then exposed to a 60-W UV lamp for 1 h. Unincorporated silver nitrate was dissolved with 5% sodium thiosulfate, and then washed with MilliQ water.

### Heterotopic bone formation assay in vivo

Eight-week-old male BALB/c nude (nu/nu) mice were obtained from Jackson Laboratory and housed in pathogen-free facilities under a 12-h light and 12-h dark cycle. hBMSCs stably infected with shDEPTOR #1, shDEPTOR #2, and shNC mixed with beta-tricalcium phosphate particles (SynthoGraft; Bicon, Boston, MA, USA) were transplanted subcutaneously under the dorsal space of the nude mice (*N* = 6 per group). The mice were sacrificed by CO_2_ asphyxiation 8 weeks after transplantation. The specimens were decalcified with 10% EDTA (pH 7.4), dehydrated, and then embedded in paraffin. Slices (5-μm thick) were prepared and stained with H&E and Masson’s trichrome. Immunohistochemical (IHC) staining using antibodies against Ocn (Osteocalcin, 1:200 dilution; Abcam, Cambridge, UK) and Deptor (1:100 dilution; Proteintech) was performed with a diaminobenzidine (DAB) Staining kit (ZSGB-BIO, Beijing, China) under the manufacturer’s instructions.

### RNA extraction, reverse transcription, and quantitative real-time PCR

Total cellular RNAs were extracted using the TRIzol reagent (Invitrogen). Reverse transcription was conducted with a PrimeScript RT Reagent Kit (Takara Bio Inc., Shiga, Japan) under the manufacturer’s instructions. Quantitative real-time PCR (qPCR) was performed with a SYBR Green Master Mix (Roche Applied Science, Mannheim, Germany). A 7500 Real-Time PCR Detection System (Applied Biosystems, Foster City, CA, USA) was used to detect gene expression, and the thermal settings used were as follows: 95 °C for 10 min, followed by 40 cycles of 95 °C for 15 s, and 60 °C for 1 min. The primers are presented in Additional file 1: Table S1, and the expression of *GAPDH* or *Gapdh* was utilized for normalization. The data were analyzed using the 2^–ΔΔCt^ relative expression method.

### Western blotting analysis

Western blotting analysis was conducted as reported previously [18]. Briefly, cells were lysed on ice with radioimmunoprecipitation assay (RIPA) buffer and a protease inhibitor cocktail (Roche Applied Science). The lysates were then centrifuged at 12000 rpm for 30 min to remove the debris. Proteins were separated by 10% SDS-PAGE and transferred to 0.45-μm polyvinylidene fluoride (PVDF) membranes. Primary antibodies against runt related transcription factor 2 (RUNX2), DEPTOR, BMP4, p-SMAD1/5/8, SMAD1 (Cell Signaling Technology, Beverly, MA, USA), OCN, and GAPDH (Abcam) were diluted 1:1000 and incubated with the membranes at 4 °C overnight. Secondary antibodies against rabbit or mouse (Cell Signaling Technology) were diluted 1:10,000 and incubated with the membranes at room temperature for 1 h. An ECL kit (CoWin Biotech) was utilized to visualize the immunoreactive proteins, and the band intensities were analyzed using ImageJ software (https://imagej.nih.gov/ij/) for quantitative calculation.

### Chromatin immunoprecipitation assay

Chromatin immunoprecipitation (ChIP) assays were performed using an EZ-Magna ChIP assay kit (Merck Millipore, Darmstadt, Germany) according to the manufacturer’s instructions. hBMSCs were seeded in 10-cm dishes, and cells were cross-linked with 1% formaldehyde after they reached 100% confluency. The cell lysate was then sonicated into DNA fragments, and the DNA–protein complexes were isolated using antibodies against DEPTOR (Merck Millipore) and isotype IgG (Cell Signaling Technology) at 4 °C overnight with constant rotation. Unbound substances were removed using elution buffer. After reversing the cross-link, the DNA was purified using spin columns, and used for quantitative ChIP-qPCR with a 7500 Real-Time PCR Detection System (Applied Biosystems). The primers specific for the *MEG3* promoter are presented in Additional file 1: Table S1. The data were calculated using the 2^–ΔΔCt^ relative expression method. Relative enrichment was then analyzed as the amount of amplified DNA normalized to the input and relative to values obtained from immunoprecipitation with isotype IgG.

### Statistical analysis

Statistical analysis was performed using SPSS Statistics 20.0 software (IBM, Armonk, NY, USA). All values were expressed as the mean ± SE from three independent experiments. Student’s *t* test was performed to analyze the differences between two groups, and for multiple comparisons a one-way analysis of variance (ANOVA) followed by Tukey’s test was conducted. *P* < 0.05 was considered statistically significant.

## Results

### High DEPTOR levels are associated with reduced bone formation in vivo

**Fig. 1:**
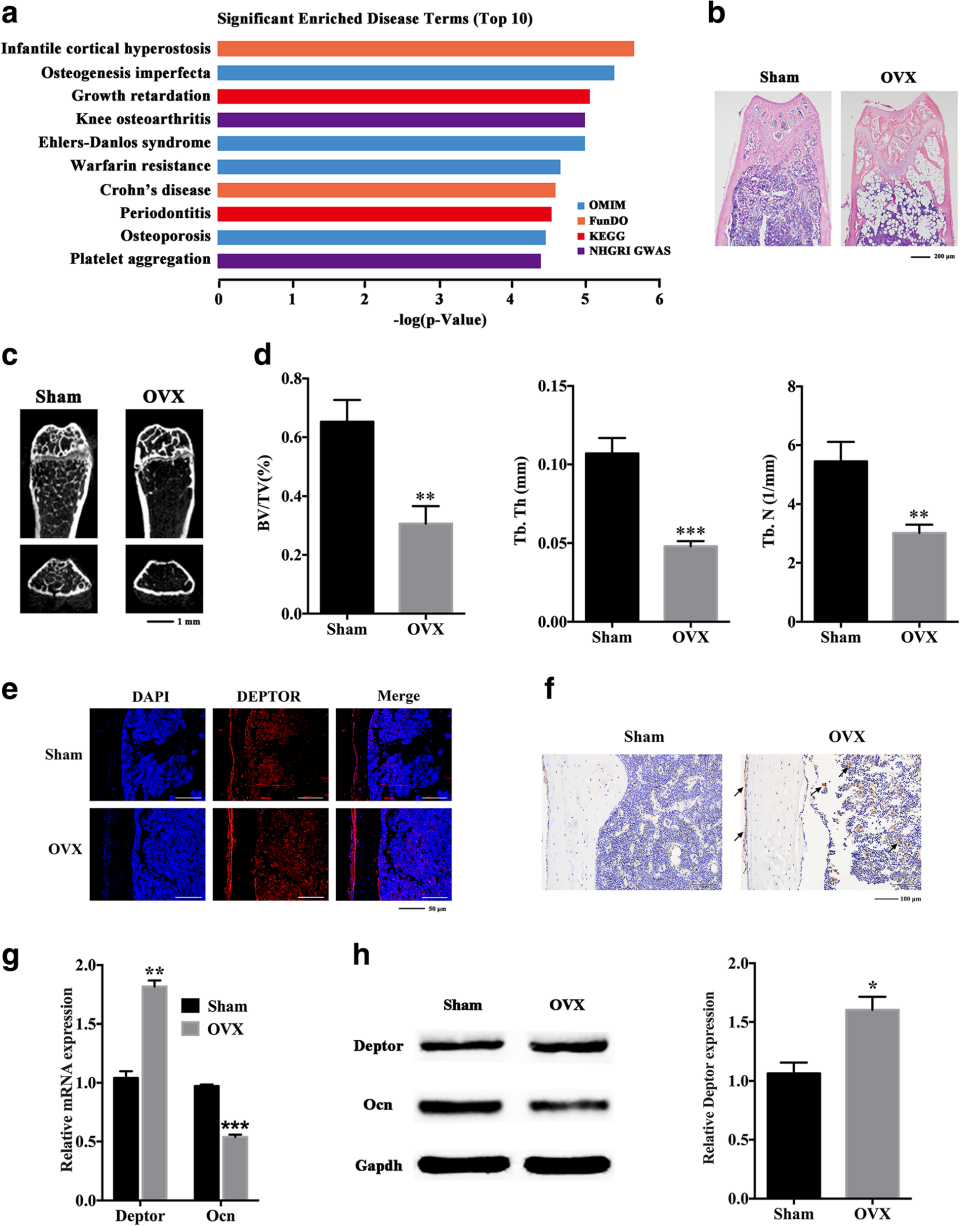
High DEPTOR levels associated with reduced bone formation in vivo. **a** Significantly enriched disease terms (top 10) associated with *DEPTOR* knockdown. **b** H&E staining of femurs at distal metaphysis growth plate area in Sham and OVX mice (*N* = 6). Scale bars: 200 μm. **c** μCT images of femurs at distal metaphysis growth plate area in Sham and OVX mice (*N* = 6). Scale bars: 1 mm. **d** Bone volume to total volume (BV/TV), trabecular bone thickness (Tb.Th), and trabecular bone number (Tb.N) reduced in OVX mice (*N* = 6). **e** Deptor expression of Sham and OVX mice evaluated by histology and immunofluorescence (*N* = 6). Scale bars: 50 μm. **f** Deptor expression of Sham and OVX mice evaluated by IHC (*N* = 6). Arrows indicate Deptor-positive areas. Scale bars: 100 μm. **g** Relative mRNA expression of *Deptor* and *Ocn* by qRT-PCR in BMSCs isolated from Sham and OVX mice. *Gapdh* used for normalization. **h** Left: western blot analysis of Deptor and Ocn protein levels in BMSCs isolated from Sham and OVX mice. Gapdh used as internal control. Right: quantification of band intensities. Data presented as mean ± SD. *P < 0.05, **P < 0.01, ***P < 0.001 (*n* = 3 independent experiments). See Additional file 2: Figure S1. DAPI 2-(4-amidinophenyl)-1H-indole-6-carboxamidine, DEPTOR DEP domain containing mTOR interacting protein, GAPDH glyceraldehyde 3-phosphate dehydrogenase, Ocn osteocalcin, OVX ovariectomy

To explore the role of DEPTOR in biological events, we established its global expression profile in BMSCs with *DEPTOR* knockdown, where NC was utilized as the scramble control. Lentiviruses expressing DEPTOR shRNA were utilized to knockdown *DEPTOR* in hBMSCs, and two shRNA sequences were generated in case of off-target effects. More than 80% of hBMSCs were GFP positive as shown in Additional file 2: Figure S1a. qRT-PCR and western blotting analysis indicated that the expression of DEPTOR was decreased significantly in the *DEPTOR* knockdown groups (Additional file 2: Figure S1b, c). According to the disease enrichment analysis, DEPTOR has a significant relationship with bone-associated diseases, such as infantile cortical hyperostosis, osteogenesis imperfecta, periodontitis, and osteoporosis (Fig. 1a). An OVX mouse model was constructed to detect the relationship between *Deptor* expression and bone formation in vivo. H&E staining, μCT images, and analyses revealed that the trabecular bone of OVX mice was dramatically reduced compared with that in the sham group (Fig. 1b–d). Immunofluorescence analyses of femur sections indicated Deptor expression both in the cortical bone and bone marrow in OVX mice in contrast to that in the sham group (Fig. 1e). Moreover, IHC analyses of Deptor indicated more positive staining both in the cortical bone and bone marrow of OVX mice compared with that in the sham mice, suggesting that the expression of Deptor was elevated in osteoporotic conditions (Fig. 1f). mBMSCs were then isolated from both sham and OVX mice. qRT-PCR and western blotting analysis demonstrated that the expression of Deptor was increased in mBMSCs from OVX mice compared with those from the sham group, while the expression of Ocn, an osteogenic marker, was significantly decreased in mBMSCs from OVX mice (Fig. 1g, h).

### DEPTOR is downregulated during the osteogenic differentiation of hBMSCs

**Fig. 2:**
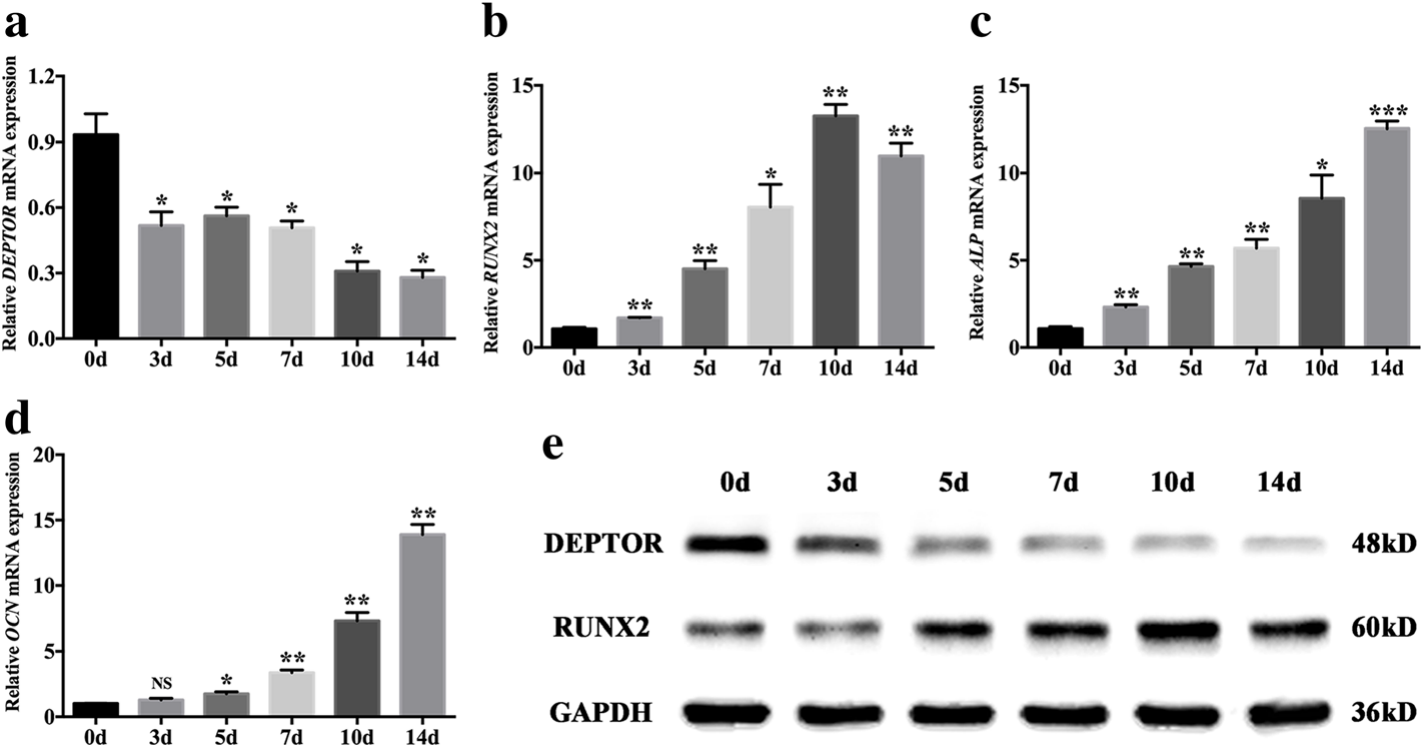
DEPTOR is downregulated in osteogenic differentiation of hBMSCs. **a** Relative expression of *DEPTOR* during osteogenic differentiation of hBMSCs determined by qRT-PCR. *GAPDH* used for normalization. **b–d** Relative mRNA levels of osteogenic markers (**b**) *RUNX2*, (**c**) *ALP*, and (**d**) *OCN* during osteogenic differentiation of hBMSCs, determined by qRT-PCR. *GAPDH* used for normalization. **e** Western blot analysis of DEPTOR and RUNX2 protein levels during osteogenic differentiation of hBMSCs. GAPDH used as internal control. Data presented as mean ± SD. *P < 0.05, **P < 0.01, ***P < 0.001, NS not significant (*n* = 3 independent experiments). ALP alkaline phosphatase, d day, DEPTOR DEP domain containing mTOR interacting protein, GAPDH glyceraldehyde 3-phosphate dehydrogenase, Ocn osteocalcin, RUNX2 runt related transcription factor 2

The expression profile of DEPTOR was determined in the osteogenic differentiation of hBMSCs to further explore its effects in vitro. As the qRT-PCR analyses indicated, downregulation of *DEPTOR* was observed during osteogenic differentiation, accompanied by upregulation of the osteogenic markers *RUNX2*, *ALP*, and *OCN* (Fig. 2a–d). Moreover, western blotting analysis demonstrated a similar decrease in the level of DEPTOR, while the level of RUNX2 increased during the osteogenic differentiation of hBMSCs (Fig. 2e).

### Knockdown of *DEPTOR* enhances osteogenic differentiation of hBMSCs in vitro

**Fig. 3:**
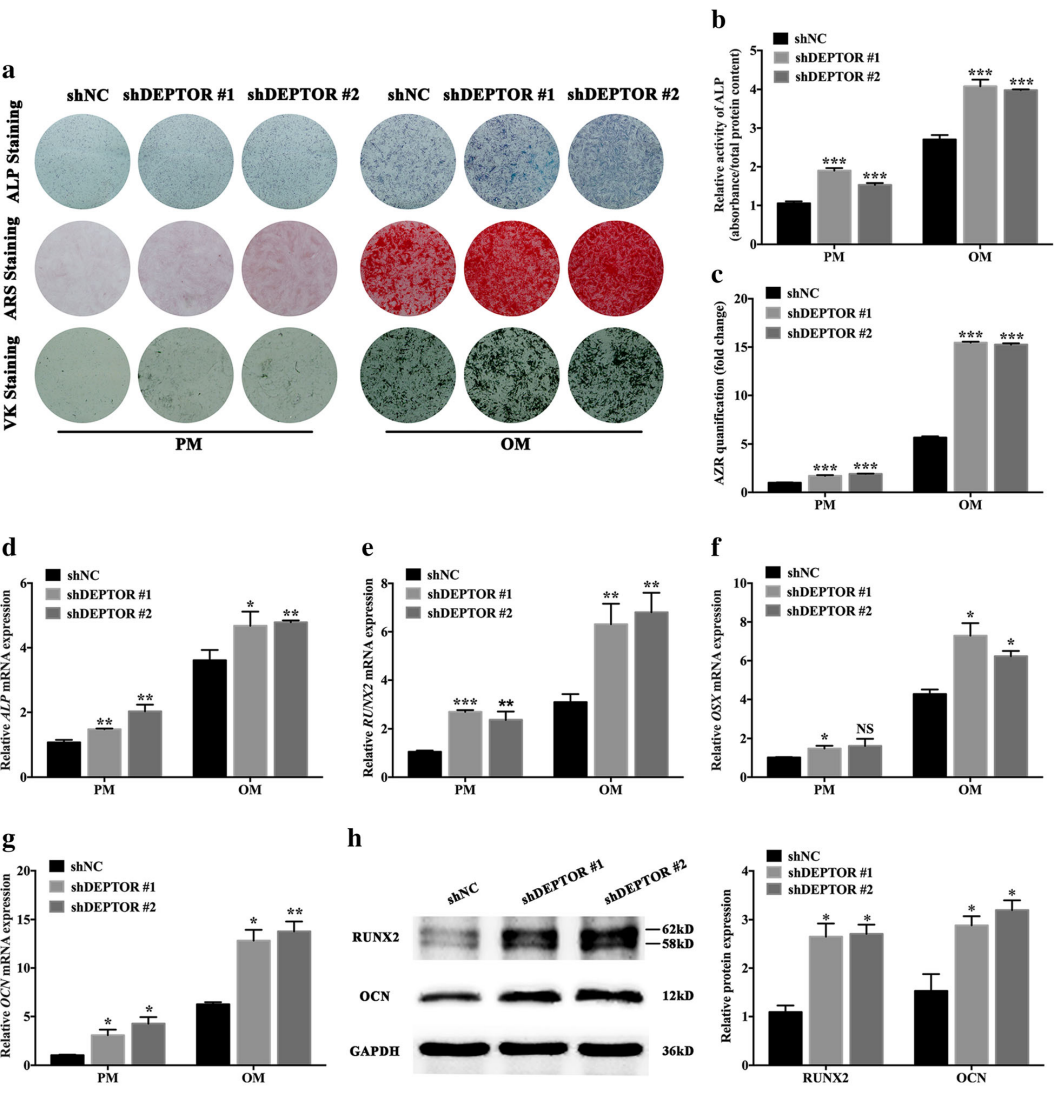
Knockdown of *DEPTOR* promotes osteogenic differentiation of hBMSCs in vitro. hBMSCs transfected with lentivirus expressing *DEPTOR* shRNAs (shDEPTOR #1, shDEPTOR #2) or scrambled control (shNC), and cultured in proliferation medium (PM) or osteogenic medium (OM). **a** ALP staining on day 7, ARS staining on day 14, and VK staining on day 21 after proliferative culture or osteogenic induction. **b, c** ALP activity on day 7 and AZR mineralization assay on day 14 after proliferative culture or osteogenic induction. **d, e** Relative mRNA levels of *ALP* and *RUNX2* measured by qRT-PCR on day 7 of proliferative culture or osteogenic induction. **f, g** Relative mRNA levels of *OSX* and *OCN* measured by qRT-PCR on day 14 of proliferative culture or osteogenic induction. *GAPDH* used for normalization. **h** Left: western blot analysis of RUNX2 and OCN protein levels on day 14 after osteogenic induction. GAPDH used as internal control. Right: quantification of band intensities. Data presented as mean ± SD. *P < 0.05, **P < 0.01, ***P < 0.001, NS not significant (*n* = 3 independent experiments). See Additional file 3: Figure S2. DEPTOR DEP domain containing mTOR interacting protein, ALP alkaline phosphatase, ARS/AZR Alizarin red S, VK von Kossa, GAPDH glyceraldehyde 3-phosphate dehydrogenase, Ocn osteocalcin, OSX osterix, RUNX2 runt related transcription factor 2

Lentiviruses were utilized to stably knockdown *DEPTOR* in hBMSCs to further investigate the effects of DEPTOR on osteogenic differentiation. Seven days after culturing the cells in PM or OM, ALP staining and quantification showed that the ALP activity was remarkably increased in the *DEPTOR* knockdown groups (Fig. 3a, b). The extracellular mineralization, as revealed by ARS staining and AZR quantification, and von Kossa staining, on day 14 and 21, respectively, was also enhanced in the *DEPTOR* knockdown groups (Fig. 3a, c). Consistently, qRT-PCR indicated that the expression levels of *RUNX2*, *ALP*, *Osterix* (*OSX*), and *OCN* were dramatically increased after *DEPTOR* knockdown in PM or OM (Fig. 3d–g). Moreover, western blotting analysis demonstrated that the protein levels of RUNX2 and OCN were elevated in the *DEPTOR* knockdown groups after osteogenic induction on day 7 and 14, respectively (Fig. 3h).

### Knockdown of *DEPTOR* promotes bone formation in vivo

Eight weeks after implantation, H&E staining showed that more osteoid tissues were formed in the *DEPTOR* knockdown group compared with the control group (Additional file 3: Figure S2a). Masson’s trichrome staining displayed more collagen deposition (blue color) after *DEPTOR* knockdown (Additional file 3: Figure S2b). Moreover, IHC staining against OCN demonstrated a higher number of brown granules around the nuclei in the *DEPTOR* knockdown groups compared with that in the control group (Additional file 3: Figure S2c).

### Knockdown of *DEPTOR* elevates MEG3 expression to regulate osteogenesis

**Fig. 4:**
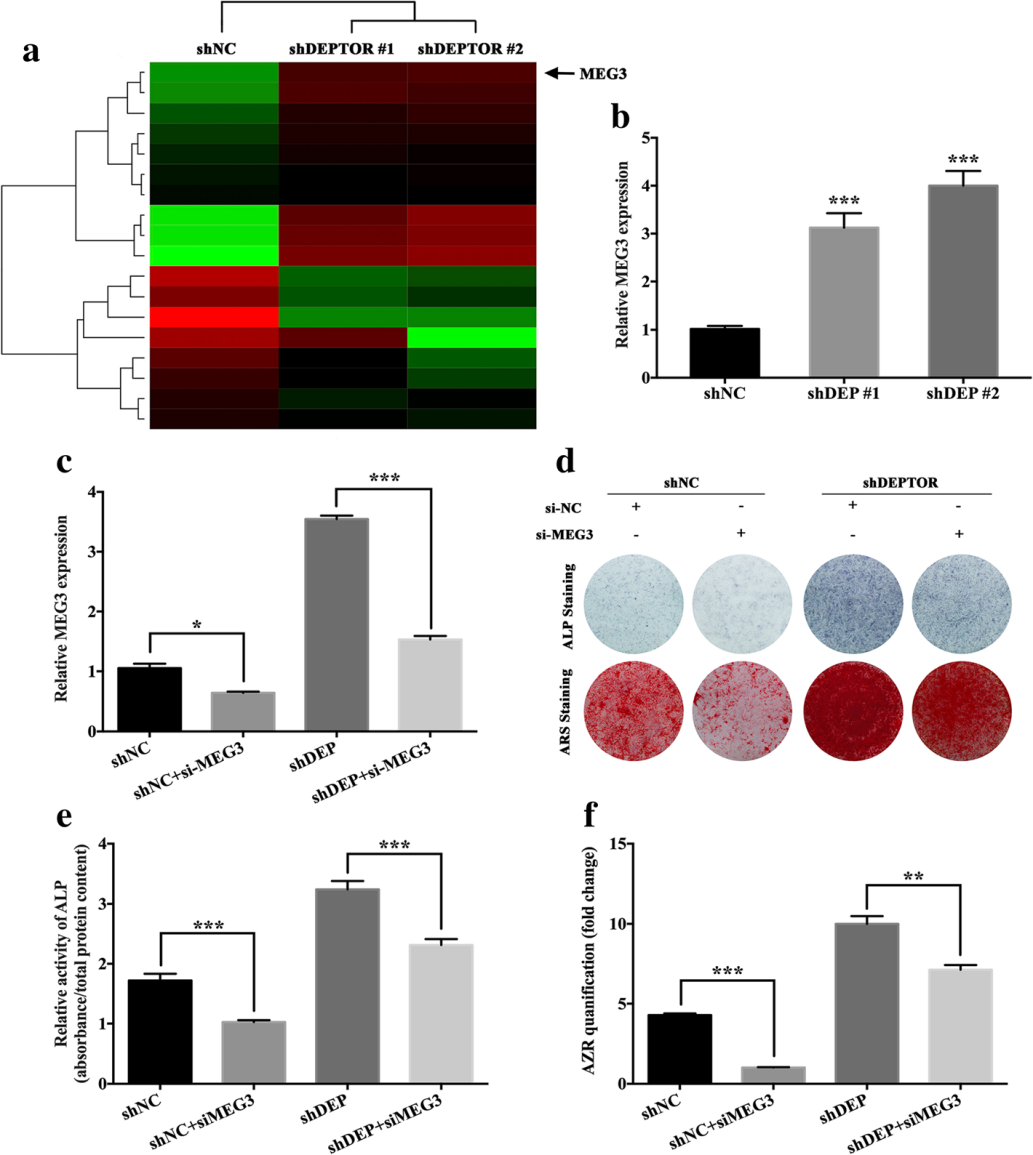
Knockdown of *DEPTOR* elevates MEG3 expression to regulate osteogenesis. **a** Hierarchical clustering indicated *MEG3* was differentially expressed (*P* < 0.05) in *DEPTOR* knockdown hBMSCs. **b** Relative *MEG3* expression measured by qRT-PCR in *DEPTOR* knockdown hBMSCs. *GAPDH* used for normalization. **c–f** *MEG3* siRNA (si-MEG3) transfected into hBMSCs with *DEPTOR* knockdown. NC used as negative control. **c** Relative *MEG3* expression determined by qRT-PCR. *GAPDH* used for normalization. **d** ALP staining on day 7 and ARS staining on day 14 after osteogenic induction. **e**, **f** ALP activity on day 7 and AZR mineralization assay on day 14 after osteogenic induction. Data presented as mean ± SD. *P < 0.05, **P < 0.01, ***P < 0.001 (*n* = 3 independent experiments). See Additional file 4: Figure S3. ALP alkaline phosphatase, ARS/AZR Alizarin red S, DEPTOR DEP domain containing mTOR interacting protein, *MEG3* maternally expressed 3 (nonprotein coding), shDEPTOR #1/shDEPTOR #2 lentivirus expressing shRNAs targeting *DEPTOR*, shNC scrambled control, sh short hairpin, si short interfering

To determine the underlying molecular mechanisms of DEPTOR in the regulation of hBMSC osteogenesis, the expression profiles of both lncRNAs and mRNAs were constructed in the *DEPTOR* knockdown groups compared with the control group. Seventy-eight lncRNAs were differentially expressed according to the threshold values of fold change ≥ 2.0 and *P* < 0.05. Among the differentially expressed lncRNAs, *MEG3* showed significant upregulation, and qRT-PCR validated that *MEG3* was increased in the *DEPTOR* knockdown cells (Fig. 4a, b). *MEG3* was demonstrated to promote osteogenic differentiation of BMSCs in recent studies [22, 23]. We further transfected *MEG3* siRNAs into hBMSCs, and the knockdown efficiency was verified using qRT-PCR (Additional file 4: Figure S3a). After culturing the cells in OM for 7 days, ALP staining and quantification indicated that knockdown of *MEG3* downregulated ALP activity (Additional file 4: Figure S3b, c). ARS staining and AZR quantification on day 14 for OM culture was also reduced in the *MEG3* knockdown groups (Additional file 4: Figure S3b, d). Moreover, to clarify whether DEPTOR regulated hBMSC osteogenesis through *MEG3*, we transfected the MEG3 siRNA into hBMSCs with *DEPTOR* knockdown, and the knockdown efficiency of *MEG3* was estimated using qRT-PCR (Fig. 4c). Seven days after osteogenic induction, ALP staining and quantification revealed that depletion of *MEG3* significantly reduced the ALP activity triggered by *DEPTOR* knockdown (Fig. 4d, e). ARS staining and AZR quantification on day 14 indicated a similar tendency (Fig. 4d, f).

### BMP4 signaling is involved in the regulation of hBMSC osteogenesis during *DEPTOR* knockdown

**Fig. 5:**
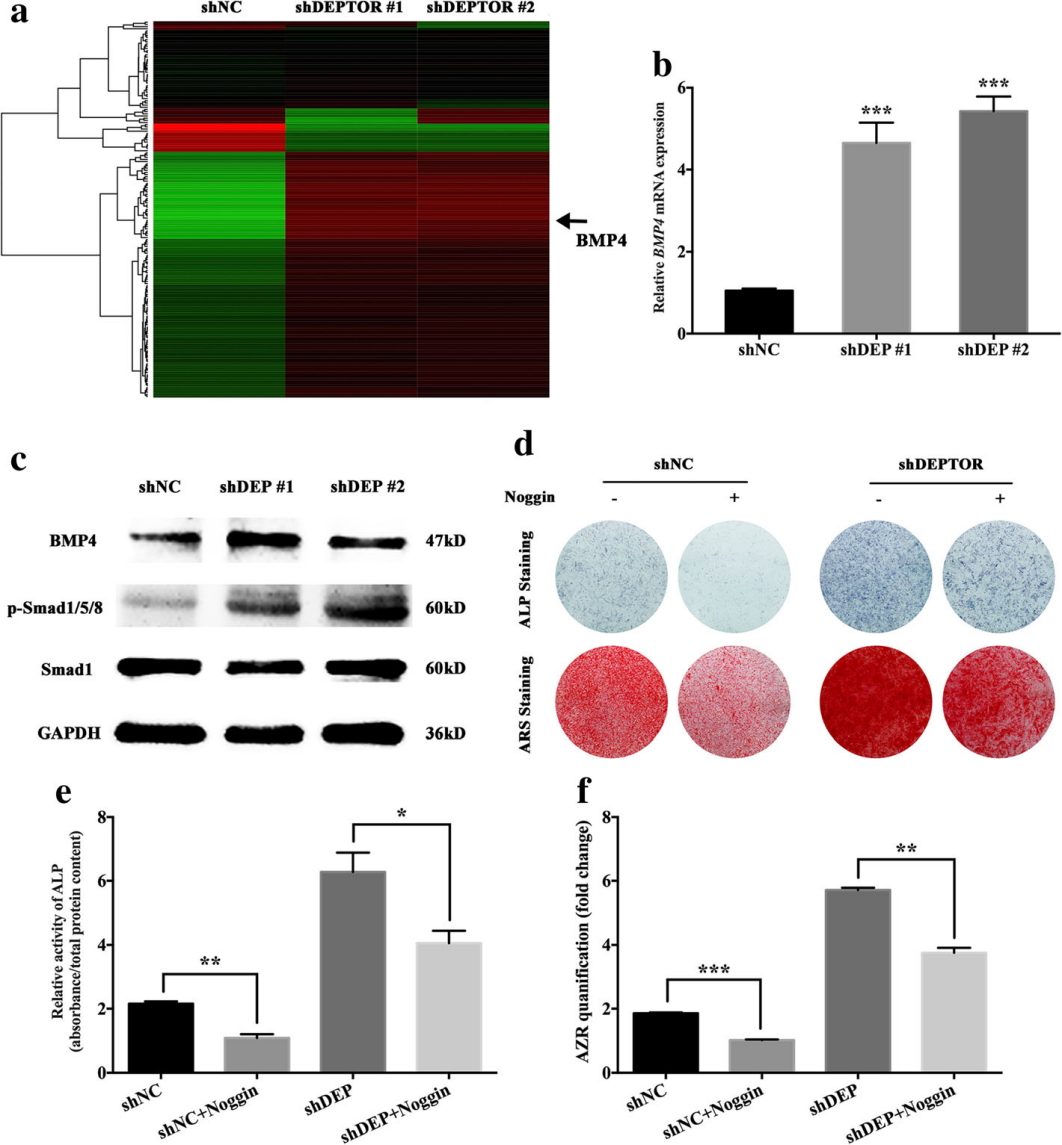
BMP4 signaling involved in regulation of hBMSC osteogenesis after *DEPTOR* knockdown. **a** Hierarchical clustering indicated *BMP4* was differentially expressed (*P* < 0.05) in *DEPTOR* knockdown hBMSCs. **b** Relative mRNA expression of *BMP4* measured by qRT-PCR in *DEPTOR* knockdown hBMSCs. *GAPDH* used for normalization. **c** Western blot analysis of BMP4 and its downstream Smad expression in *DEPTOR* knockdown hBMSCs. GAPDH utilized as internal control. **d–f** BMP antagonist Noggin (500 ng·mL^− 1^) administered to *DEPTOR* knockdown and NC hBMSCs. **d** ALP staining on day 7 and ARS staining on day 14 after osteogenic induction. **e**, **f** ALP activity on day 7 and AZR quantification on day 14 after osteogenic induction. Data presented as mean ± SD. *P < 0.05, **P < 0.01, ***P < 0.001 (*n* = 3 independent experiments). ALP alkaline phosphatase, ARS/AZR Alizarin red S, BMP4 bone morphogenetic protein 4, DEPTOR DEP domain containing mTOR interacting protein, GAPDH glyceraldehyde 3-phosphate dehydrogenase, shDEPTOR #1/shDEPTOR #2 lentivirus expressing shRNAs targeting *DEPTOR*, shNC scrambled control, sh short hairpin

We identified 1107 mRNAs that were differentially expressed between *DEPTOR* knockdown and control cells, according to the thresholds values of fold change ≥ 2.0 and *P* < 0.05. Among the differentially expressed mRNAs, genes within the BMP signaling pathway were dramatically upregulated in *DEPTOR* siRNA cells, in which the upstream *BMP4* gene showed a remarkable increase (Fig. 5a). qRT-PCR and western blotting analysis indicated that knockdown of *DEPTOR* increased the expression of *BMP4*, leading to more phosphorylated SMAD1/5/8, while the total SMAD1 remained barely changed (Fig. 5b, c). Next, we treated *DEPTOR* knockdown hBMSCs with the BMP antagonist Noggin. ALP staining and quantification after osteogenic induction for 7 days indicated that delivery of Noggin dramatically attenuated the ALP activity in the *DEPTOR* knockdown hBMSCs (Fig. 5d, e). Similar outcomes were observed for ARS staining and AZR quantification on day 14 after osteogenic differentiation (Fig. 5d, f).

### Knockdown of *DEPTOR* activates BMP4 signaling through MEG3

**Fig. 6:**
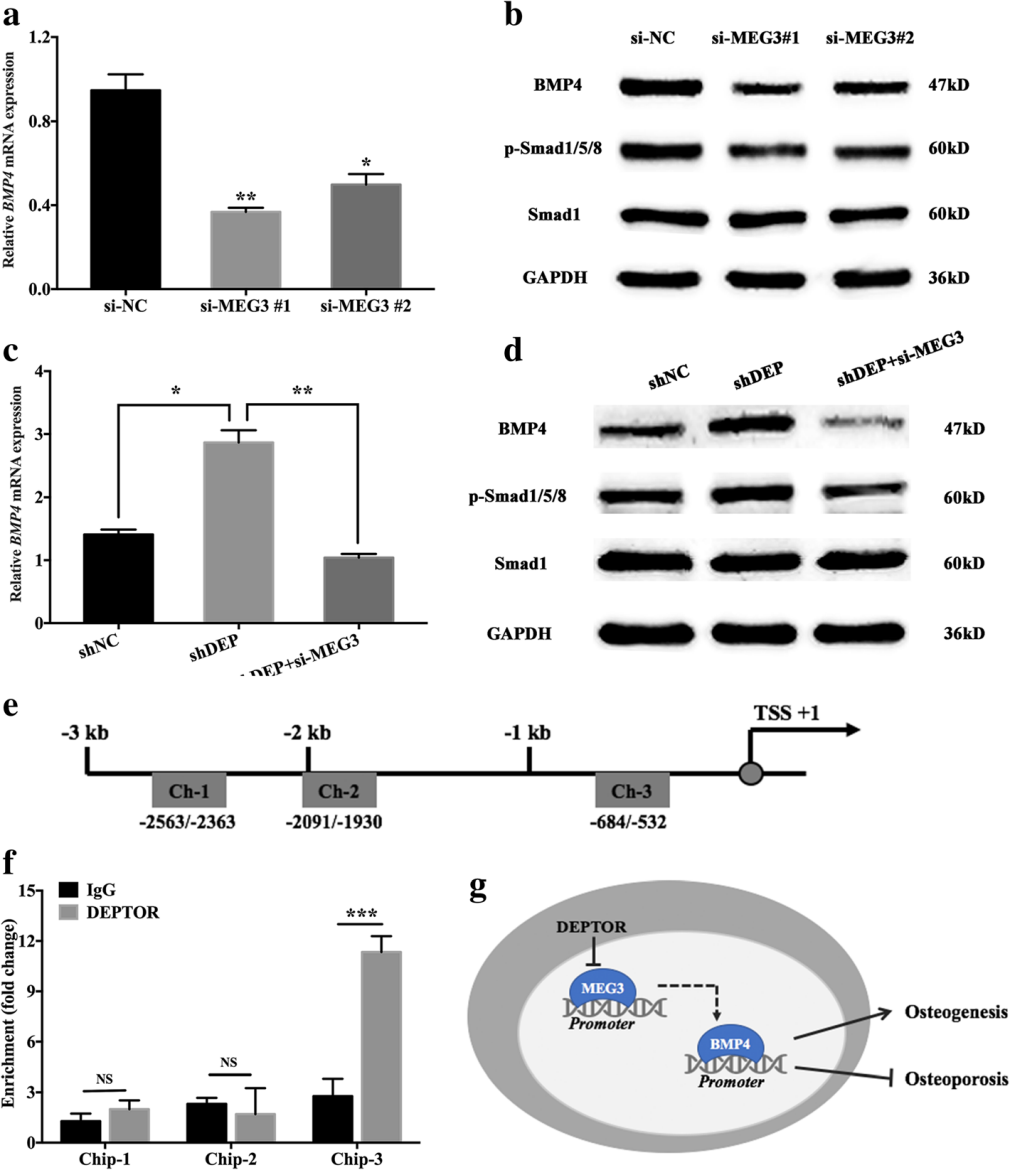
Knockdown of *DEPTOR* activates BMP4 signaling through MEG3. **a** Relative mRNA expression of *BMP4* in *MEG3* knockdown (si-MEG3 #1, si-MEG3 #2) and control vector (si-NC) groups. *GAPDH* used for normalization. **b** Western blot analysis of BMP4 and its downstream Smad expression in *MEG3* knockdown and NC groups. GAPDH utilized as internal control. **c, d** *MEG3* siRNA (si-MEG3) introduced into *DEPTOR* knockdown hBMSCs. **c** Relative mRNA expression of *BMP4* measured using qRT-PCR. *GAPDH* used for normalization. **d** Western blot analysis of BMP4 and its downstream Smad expression. GAPDH utilized as internal control. **e** *MEG3* promoter and location of primers. Positions marked relative to the transcription start site (TSS). **f** ChIP-qPCR showing interaction between DEPTOR and *MEG3* promoter in hBMSCs. IgG used for normalization. Data presented as mean ± SD. *P < 0.05, **P < 0.01, ***P < 0.001, NS not significant (*n* = 3 independent experiments). **g** Proposed mechanism of DEPTOR modulation of osteogenesis via regulation MEG3-mediated BMP4 activation. BMP4 bone morphogenetic protein 4, Chip chromatin immunoprecipitation, DEPTOR DEP domain containing mTOR interacting protein, GAPDH glyceraldehyde 3-phosphate dehydrogenase, *MEG3* maternally expressed 3 (nonprotein coding), shNC scrambled control, sh short hairpin, si short interfering

Previous research demonstrated that MEG3 could modulate *BMP4* transcription through directly interacting with the core element of the *BMP4* promoter [22]. In the present study, we detected *BMP4* expression in hBMSCs transfected with *MEG3* siRNAs. As the qRT-PCR analysis indicated, the mRNA level of *BMP4* was reduced in the *MEG3* knockdown groups (Fig. 6a). Moreover, western blotting analysis indicated that knockdown of *MEG3* in hBMSCs inhibited BMP4 signaling, which was evidenced by the decrease in the BMP4 level, and the subsequent attenuation of phosphorylated SMAD1/5/8 (Fig. 6b). To further clarify whether DEPTOR modulates BMP4 signaling through MEG3, we established *DEPTOR* and *MEG3* double knockdown cells. qRT-PCR indicated that depletion of *MEG3* reduced the *BMP4* expression in the *DEPTOR* knockdown group (Fig. 6c) compared with that in the control group. Moreover, western blotting analysis showed that BMP4 and its downstream phosphorylated SMAD1/5/8 were also decreased in the *DEPTOR* and *MEG3* double knockdown cells compared with that in the *DEPTOR* knockdown cells (Fig. 6d). DEPTOR might function as a transcriptional regulator; therefore, we further investigated whether DEPTOR was directly responsible for *MEG3* transcription [24]. Sequence analysis of the *MEG3* promoter, comprising − 3000 bp upstream from the transcription start site (TSS), demonstrated three potential binding sites for DEPTOR (Fig. 6e). ChIP-qPCR showed that DEPTOR could bind to the specific region of the *MEG3* promoter, with dramatic enrichment in the third region (− 1000 bp ~ 0) (Fig. 6f).

## Discussion

Recent studies indicated that the abnormal lineage commitment of BMSCs is closely related to the bone loss and fat accumulation in bone marrow during osteoporosis [5]. BMSCs derived from elderly people have a decreased tendency to differentiate into osteoblasts, accompanied with an increased tendency toward the adipogenic lineage [25, 26]. As part of the *Fob3a* quantitative trait locus (QTL), a gene that was linked to obesity/leanness, *Deptor*, was involved in adipogenic regulation [27]. Our disease enrichment analysis revealed that DEPTOR might be associated with osteoporosis. In the present study, we used OVX mice as an osteoporotic model, which has been commonly recognized as a model of BMSCs with low osteogenic capability [19, 21, 28]. Elevated expression of Deptor was observed in both trabecular bones and BMSCs of osteoporotic mice, indicating that DEPTOR might play an important role during the progress of osteoporosis.

The osteoblastic and adipocytic lineages have been recognized as alternatives during cell differentiation [6, 7]. DEPTOR has been established as a positive regulator of adipogenesis. The expression of Deptor in white adipose tissue (WAT) correlated with the degree of obesity, and its overexpression accumulated more WAT in mice [17], while its effects in the osteogenic differentiation of BMSCs remain elusive. We demonstrated that DEPTOR expression was consistently decreased as BMSCs committed to the osteoblastic lineage. Moreover, knockdown of *DEPTOR* via lentivirus transfection enhanced the osteogenic differentiation of BMSCs both in vitro and in vivo, indicating DEPTOR as a novel regulator of osteogenesis.

The negative effects of DEPTOR in regulating osteogenesis seem counterintuitive, considering that DEPTOR was reported as an endogenous inhibitor of mTOR, which has controversial effects on the osteogenic differentiation of BMSCs [11, 12]. Functioning in two distinct multiprotein complexes, mTORC1 mainly phosphorylates S6 K1 and 4E-BP1 to control protein synthesis, whereas mTORC2 phosphorylates AKT at Ser 473 to modulate cell survival [9, 29]. It has been reported that deletion of S6 K1 enhanced resistance to age-related bone loss, while ablation of Rictor reduced the osteogenic differentiation of BMSCs [30, 31]. A recent study using Cre-mediated deletion of *Rptor* (RapKO) or *Rictor* (RicKO) revealed different roles of mTORC1 and mTORC2 in the osteogenic differentiation of MSCs. With impaired mTORC1 signaling, RapKO MSCs displayed an increased capacity for osteogenesis, while RicKO MSCs demonstrated a reduced capacity for osteogenesis in the presence of limited mTORC2 signaling [12]. This can be accounted for the confounding result that suppression of mTOR signaling with rapamycin had both stimulatory and inhibitory osteogenic effects [32, 33]. As the research showed, rapamycin primarily inhibited mTORC1 activity to promote osteogenesis, while prolonged exposure disrupted mTORC2 function to inhibit osteogenesis [34, 35]. Our previous study demonstrated that knockdown of *DEPTOR* activated S6 K1, which subsequently enhanced the negative feedback loop on IRS1/PI3K to inhibit AKT signaling, whereas the activity of mTOR remained inactivated as the phosphorylated mTOR was reduced by *DEPTOR* knockdown [18]. The insufficient activation of mTOR prompted further investigation of how DEPTOR regulates osteogenic differentiation.

Recent studies indicated that, independent of mTOR inhibitors, DEPTOR could function as a transcriptional regulator, and binds to specific promoter regions of genes to regulate endoplasmic reticulum homeostasis, such as *PSEN2*, *CKAP4*, and *KEAP1* [24]. Recently, lncRNAs were observed to play important roles in the differentiation of MSCs, and high-throughput gene sequencing revealed that more than 1000 differentially expressed lncRNAs were identified during the osteogenic differentiation of BMSCs [36, 37]. We established an RNA-seq analysis to determine the expression profiles of both lncRNAs and mRNAs with *DEPTOR* knockdown in BMSCs, and 78 lncRNAs and 1107 mRNAs were differentially expressed. Among the differentially expressed lncRNAs, *MEG3* has been implicated in the osteogenic differentiation of MSCs [22, 23, 38]. As a maternally expressed imprinted gene representing an lncRNA, *MEG3* is widely recognized as a tumor suppressor that is expressed at low levels in some cancers and correlates with prognosis [39, 40]. Previous research demonstrated that *MEG3* promoted osteogenic differentiation of MSCs from patients with multiple myeloma, and our recent study displayed a similar promotion of osteogenesis on hASCs with *MEG3* overexpression [22, 23]. However, another study of postmenopausal osteoporosis revealed that *MEG3* inhibited the osteogenic differentiation of BMSCs [38]. In the present study, we found that inhibition of DEPTOR remarkably upregulated the expression of *MEG3*, and further investigation indicated that DEPTOR could directly bind to a specific region (− 1000 bp ~ 0) of the *MEG3* promoter to regulate its transcription. Moreover, inhibition of *MEG3* reduced the enhancement of osteogenic differentiation triggered by *DEPTOR* knockdown, suggesting that DEPTOR regulated BMSC osteogenesis through *MEG3*.

lncRNAs participate in gene expression by regulating the transcriptional activity of neighboring coding regions, and can enhance gene expression over several Mbs [41, 42]. Previous research has shown that *MEG3* can dissociate the transcriptionally inhibitory factor SOX2 from the *BMP4* promoter to enhance *BMP4* expression [22]. Here we revealed that knockdown of *DEPTOR* increased BMP4 expression, which subsequently phosphorylated Smad1/5/8 to activate Smad signaling. Furthermore, when treated with Noggin, a BMP4 antagonist, BMSCs with *DEPTOR* knockdown exhibited reduced capacity toward osteogenesis. As a member of the transforming growth factor beta (TGF-β) family, BMP4 was identified as a regulator of bone and cartilage formation [43]. Emerging evidence indicates that BMP signaling plays a pivotal role in osteogenic differentiation and bone formation [44, 45]. Given the relationship between MEG3 and BMP4, we speculated that knockdown of *DEPTOR* could activate BMP4 signaling through MEG3. As the results suggested, depletion of *MEG3* reduced the activation of BMP4 signaling triggered by *DEPTOR* knockdown.

## Conclusions

In this study, we determined the functions of DEPTOR in the osteogenic differentiation of BMSCs using a cellular biology approach and an osteoporotic animal approach that represents a BMSC model with low osteogenic capability. Our findings increased our understanding of the regulatory mechanisms of the osteogenic differentiation of BMSCs, and might contribute to bone tissue engineering and the treatment of osteoporotic diseases.

## Additional files

Additional file 1: **Table S1.** Sequences of RNA and DNA oligonucleotides. (DOCX 17 kb)

Additional file 2: **Figure S1.** Transduction efficiency of lentivirus expressing DEPTOR shRNAs (shDEPTOR #1, shDEPTOR #2) or scrambled control (shNC) in hBMSCs. (TIF 1443 kb)

Additional file 3: **Figure S2.** Knockdown of *DEPTOR* promotes bone formation in vivo. (TIF 1782 kb)

Additional file 4: **Figure S3.** Knockdown of *MEG3* reduces osteogenic differentiation of hBMSCs. (TIF 1035 kb)

## Abbreviations

ALP: Alkaline phosphatase
ANOVA: Analysis of variance
ARS/AZR: Alizarin red S
ASBMR: American Society for Bone and Mineral Research
BMSC: Bone marrow mesenchymal stem cell
BV/TV: Bone volume/total volume
DEPTOR: DEP domain containing mTOR interacting protein
ESC: Embryonic stem cell
FBS: Fetal bovine serum
FDR: False discovery rate
FPKM: Fragments per kilobase of exon per million fragments mapped
H&E: Hematoxylin and eosin
hBMSC: Human bone marrow mesenchymal stem cell
IHC: Immunohistochemical
mBMSC: Mouse bone marrow mesenchymal stem cell
mTOR: Mammalian target of rapamycin
OCT: Optimum cutting temperature
OM: Osteogenic medium
OSX: Osterix
OVX: Ovariectomy
PM: Proliferation medium
qRT-PCR: Quantitative real-time reverse transcription PCR
QTL: Quantitative trait locus
Tb.N: Trabecular bone number
Tb.Th: Trabecular bone thickness
TGF-β: Transforming growth factor beta
TSS: Transcription start site
WAT: White adipose tissue
α-MEM: Minimum essential medium alpha
