# Supplementary material for: DEPTOR regulates osteogenic differentiation via inhibiting MEG3-mediated activation of BMP4 signaling and is involved in osteoporosis
Source: Stem Cell Res Ther. 2018 Jul 4;9:185. doi: 10.1186/s13287-018-0935-9 (PMC6033203; doi:10.1186/s13287-018-0935-9)
Supplement: Supplementary file 1 — Table S1. Sequences of RNA and DNA oligonucleotides. (DOCX 17 kb) [file 13287_2018_935_MOESM1_ESM.docx]

**Table S1:** Sequences of RNA and DNA oligonucleotides

| **Name** | **Sense Strand/Sense Primer (5'-3')** | **Antisense Strand/Antisense Primer (5'-3')** |  |
| --- | --- | --- | --- |
| **siRNA** | | | |
| MEG3 #1 | GCUCAUACUUUGACUCUAUTT | AUAGAGUCAAAGUAUGAGCTT |  |
| MEG3 #2 | CCCUCUUGCUUGUCUUACUTT | AGUAAGACAAGCAAGAGGGTT |  |
| NC | UUCUCCGAACGUGUCACGUTT | ACGUGACACGUUCGGAGAATT |  |
| **shRNA**  DEPTOR #1 GTCATCATCTCAAGACCTA  DEPTOR #1 GTCTGTCAGTTTGTCGTCT  NC TTCTCCGAACGTGTCACGT  **Primers for qRT-PCR** | | | |
| *RUNX2* | CCGCCTCAGTGATTTAGGGC | GGGTCTGTAATCTGACTCTGTCC |  |
| *ALP* | ATGGGATGGGTGTCTCCACA | CCACGAAGGGGAACTTGTC |  |
| *OSX* | CCTCTGCGGGACTCAACAAC | TAAAGGGGCTGGATAAGCAT |  |
| *OCN* | CACTCCTCGCCCTATTGGC | CCCTCCTGCTTGGACACAAAG |  |
| *DEPTOR* | TTTGTGGTGCGAGGAAGTAA | CATTGCTTTGTGTCATTCTGG |  |
| *MEG3*  *BMP4* | GCCCTGACCTTTGCTATGCT  ATGATTCCTGGTAACCGAATGC | TCGACAAAGACTGACACCCC  CCCCGTCTCAGGTATCAAACT |  |
| *GAPDH*  *Deptor*  *Ocn*  *Gapdh* | GAAGGTGAAGGTCGGAGTC  AGCAGAGAGAGCTGGAACGC  CACTCCTCGCCCTATTGGC  ACAGCAACTCCCACTCTTCCAC | GAAGATGGTGATGGGATTTC  CAGAGGCCTCCTTATGTTCA  CCCTCCTGCTTGGACACAAAG  AGTTGGGATAGGGCCTCTCTTG |  |
| **Primers for Chip-qPCR**  Chip-1 TGTGCCAAGGACTTGTGC TTTCGGAGGTCAGGATGAG  Chip-2 GCCTGGTGGTGTCTTCTTTC CACAGCCTTGCCTTAGTTCC  Chip-3 GAGCCGACTGACATGGAGA AGGCAGGGAGAAGGAGGA | | |  |
