# Supplementary figures and images for: DEPTOR regulates osteogenic differentiation via inhibiting MEG3-mediated activation of BMP4 signaling and is involved in osteoporosis
Source: Stem Cell Res Ther. 2018 Jul 4;9:185. doi: 10.1186/s13287-018-0935-9 (PMC6033203; doi:10.1186/s13287-018-0935-9)

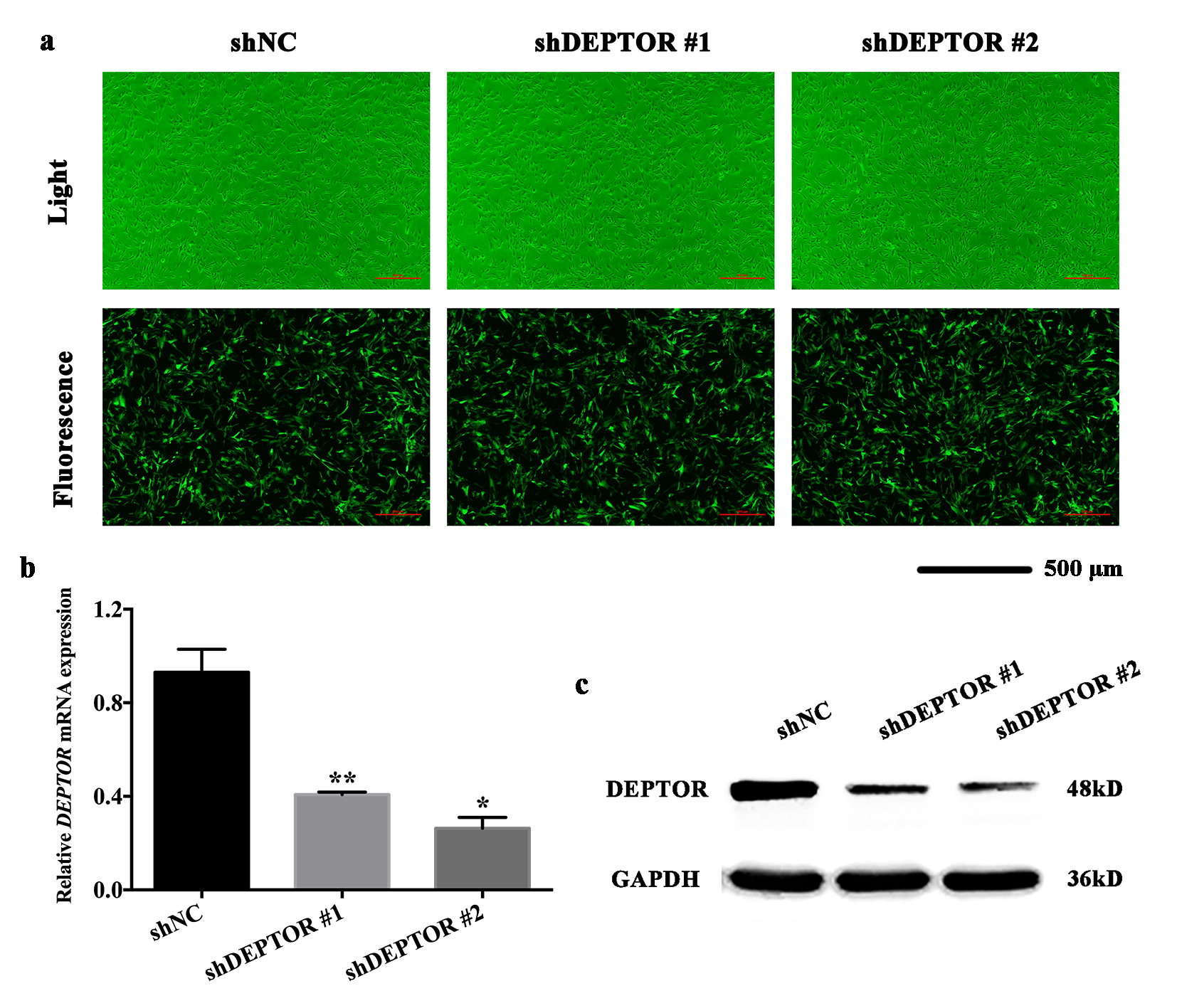

Supplement: Supplementary file 2 — Figure S1. Transduction efficiency of lentivirus expressing DEPTOR shRNAs (shDEPTOR #1, shDEPTOR #2) or scrambled control (shNC) in hBMSCs. (TIF 1443 kb) [file 13287_2018_935_MOESM2_ESM.tif]

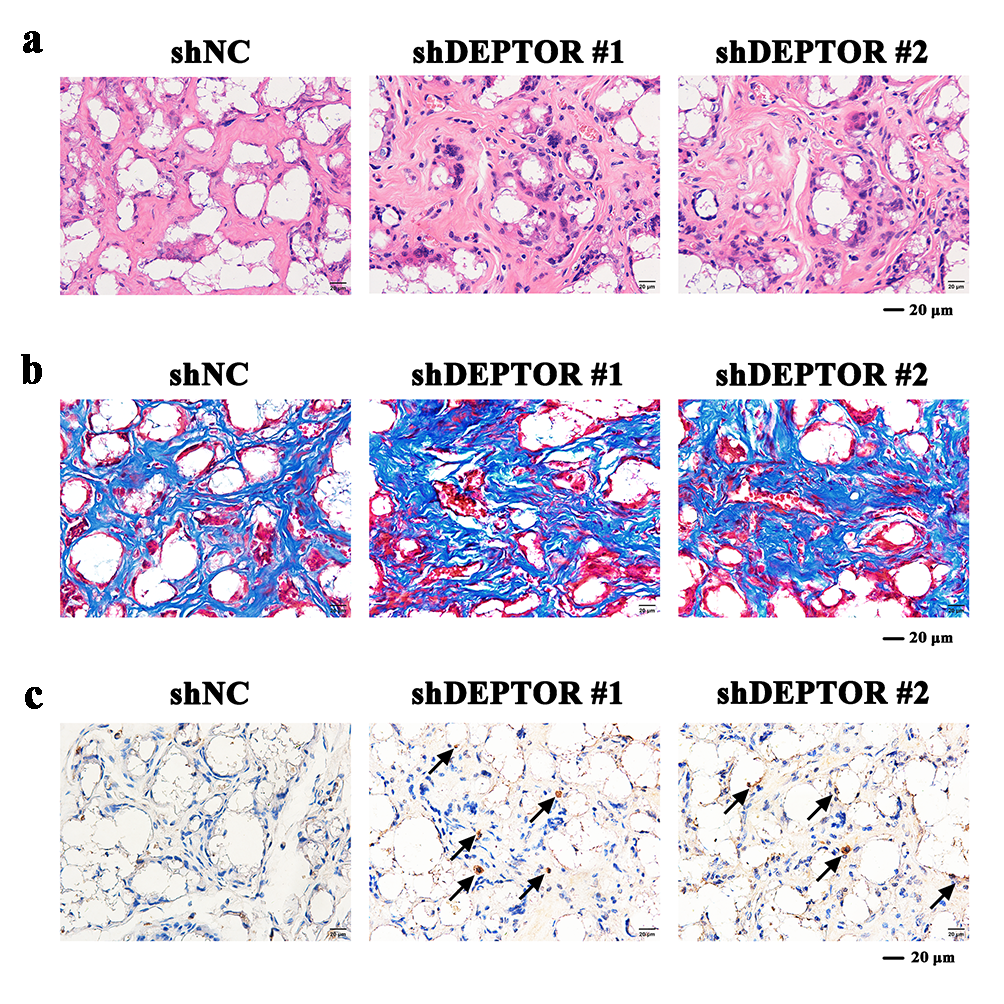

Supplement: Supplementary file 3 — Figure S2. Knockdown of DEPTOR promotes bone formation in vivo. (TIF 1782 kb) [file 13287_2018_935_MOESM3_ESM.tif]

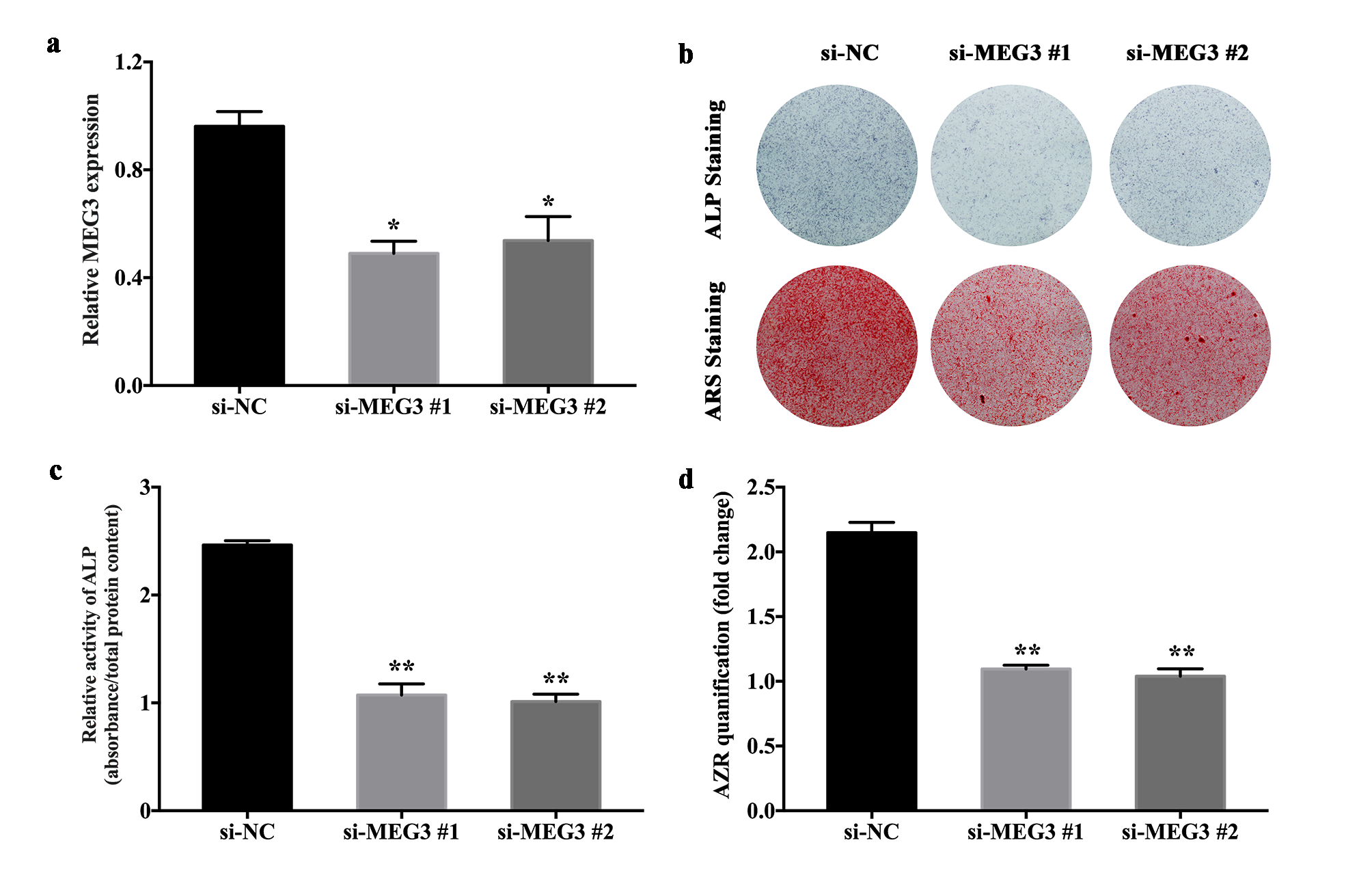

Supplement: Supplementary file 4 — Figure S3. Knockdown of MEG3 reduces osteogenic differentiation of hBMSCs. (TIF 1035 kb) [file 13287_2018_935_MOESM4_ESM.tif]
